# Clinical utility of Thromboelastography in patients with COVID-19: a prospective observational study

**DOI:** 10.1186/s12873-025-01381-y

**Published:** 2025-11-10

**Authors:** A. Deepak Sai, Ravindra Prithvishree, K. Nagesh Savan, Mohan Ganesh, Gupta Nitin, Kishore Asish, S. Nisarg, Chaudhuri Souvik, Paonam Bemma

**Affiliations:** 1 Department of Emergency Medicine, Kasturba Medical College, Manipal, Manipal Academy of Higher Education, Manipal, Karnataka India; 2 Department of Anesthesiology, Kasturba Medical College, Manipal, Manipal Academy of Higher Education, Manipal, Karnataka India; 3 Department of Immunohematology and Blood Transfusion, Kasturba Medical College, Manipal, Manipal Academy of Higher Education, Manipal, Karnataka 576104 India; 4 Department of Infectious Diseases, Kasturba Medical College, Manipal, Manipal Academy of Higher Education, Manipal, Karnataka India; 5 Department of Critical Care Medicine, Kasturba Medical College, Manipal, Manipal Academy of Higher Education, Manipal, Karnataka India

**Keywords:** TEG, COVID-19, COVID-associated coagulopathy, Thrombosis, Hyper coagulopathy

## Abstract

**Introduction:**

Coronavirus disease (COVID-19) has been associated with thromboembolic and bleeding complications. Our study aims to understand the utility of early Thromboelastography (TEG) in the emergency department as a point-of-care test in predicting clinical outcomes.

**Materials and methodology:**

A prospective observational study was conducted in the emergency medicine department (ED) during June 2021- May 2022 with inclusion criteria of all patients above 18 years who arrived at the ED and had a positive COVID-19 RT PCR test. Hematological investigation, including TEG, was sent. Patients were followed up for 28-day mortality, thrombotic and bleeding complications.

**Results:**

A total of 166 patients were enrolled in the study. 46% of patients had critical COVID-19. TEG was abnormal in 45% of patients. Hypercoagulability was seen in 32% of patients. 28-day mortality in our study population was 32.3%. The most common thrombotic complications were stroke (4.2%), myocardial infarction (3.6%), pulmonary embolism (1.2%), and DVT (0.6%). Logistic regression analysis showed that patients who had hypocoagulable TEG had a higher 28-day mortality.

**Conclusion:**

COVID-associated coagulopathy can be associated with both thrombotic (venous and arterial) and bleeding complications. Hypocoagulability on initial TEG has a 7.49 times increased risk of mortality. Hypocoagulable TEG indicates an early predictor for mortality. Early TEG can be an essential tool in managing COVID-associated coagulopathy.

**Supplementary Information:**

The online version contains supplementary material available at 10.1186/s12873-025-01381-y.

## Introduction

Coronavirus disease (COVID-19) is caused by severe acute respiratory syndrome coronavirus 2 (SARS-CoV-2). Since the diagnosis of the first case in December 2019, it has rapidly spread across the globe, leading to a profound public health problem, claiming millions of lives [1]. It primarily affects the respiratory system and presents as fever, cough, and shortness of breath. Additionally, it can also present as extrapulmonary manifestations, including cardiovascular, neurological, and thromboembolic complications [2]. Hypercoagulopathy in COVID-19 leads to thromboembolic complications such as pulmonary thromboembolism, deep vein thrombosis, stroke, and acute coronary syndrome. It affects multiple organs and can cause long term effects, which may lead to lifestyle modifications [3]. 

Thromboelastography (TEG) is a whole blood-based viscoelastic test used to evaluate the dynamic properties of blood coagulation. It provides a comprehensive analysis of the coagulation process by measuring interactions between plasma coagulation factors and cellular factors. Conventional coagulation tests like PT, APTT, platelet count, and fibrinogen concentration take significant time (45–60 min) and only measure individual components of the coagulation cascade, but TEG gives a real-time assessment of the entire coagulation process, including clot initiation, propagation, stabilization, and dissolution in lesser time (15–30 min). Detecting a hypercoagulable coagulation status is another advantage of TEG over conventional coagulation tests. It has been proven to be a valuable tool in managing coagulopathies in various clinical settings, including surgery, trauma, and liver disease [4].

The usefulness of TEG in COVID-19 has gained attention due to a predisposition for thromboembolic complications in COVID-19. COVID-19-associated coagulopathy (CAC) is characterized by a thrombotic condition predominantly observed in early phases of infection, with a cumulative incidence of thromboembolic events in CAC is around 30% [5, 6]. TEG could significantly identify hypercoagulability in COVID-19 patients, guide anticoagulant therapy, and improve clinical outcomes. The severe inflammatory state secondary to the infection leads to a derangement of hemostasis, typically observed in patients with sepsis and has been described as a state of acute disseminated intravascular coagulation (DIC) [7]. As the severity of the disease advances, a hypocoagulable picture is also observed with a hemorrhagic tendency because of widespread endotheliopathy, consumption of coagulation factors, and acidosis [8]. A systematic review by Hartman et al. demonstrated that TEG helps identify coagulation abnormalities earlier than standard tests, thus providing critical insights for tailored therapeutic interventions [9].

TEG monitors dynamic changes in coagulation parameters, which makes it helpful in managing COVID-19. As COVID-19 causes coagulation abnormalities that can lead to both thrombosis and bleeding manifestations, TEG can be a pivotal tool in the diagnostic and therapeutic arsenal against this multifaceted disease. Our study aims to understand the possible utility of early TEG in the emergency department as a point-of-care test predicting clinical outcomes such as thromboembolism, bleeding, and mortality.

## Methodology

We conducted a prospective observational study in a tertiary care center in South India from June 2021 to May 2022. Approval from the Institutional Ethics Committee was obtained, and the trial was registered under CTRI [CTRI/2021/09/0362275] before the recruitment of patients. Assuming the prevalence of hyper coagulopathy among COVID-19 patients as 31% (Salem et al.) [10] with a confidence interval of 95% and a margin of error of 7.5%, the estimated sample size was 147 patients.

All patients above 18 years who arrived in the Emergency Department and were confirmed COVID cases by a positive COVID-19 RT PCR (nasal or throat) during the study period were included. Patients with pre-existing coagulation disorders were excluded.

The data was collected after obtaining consent from the patient or their legally acceptable representatives. Demographic data of all the included patients, including age, comorbidities, and medications, were documented after interviewing the patient or their relatives. Patient’s vital signs [heart rate, systolic and diastolic blood pressure, room air saturation], requirement of oxygen support, and presence of shock at admission were noted. Hematological investigations, such as Complete blood count, D-dimer, Prothrombin time, Activated Partial Thromboplastin Time, and TEG [TEG 500, Hemonetics, USA] were sent at the time of admission, the results of which were recorded. The NIAID ordinal scoring system was documented at admission and at the time of final disposition of the patient. Charlson’s comorbidity index was calculated. Treatment during the hospital stay was recorded. Based on the history from the interview, the patients with pre-existing coagulation disorders were excluded.

The outcomes of the patients were recorded as death, discharge, or discharge against medical advice. Duration of hospital stay and number of days of ICU stay (if any) were also calculated. The presence of thrombotic complications was considered if the patients had a new onset of Myocardial infarction as defined by the fourth universal definition of myocardial infarction [11], or had imaging evidence of deep vein thrombosis, acute pulmonary thromboembolism, or stroke. Thromboembolism risk estimation was assessed by the serial Doppler and Echo. They were done on admission and on day 5 or when it was feasible. The presence of Upper gastrointestinal bleeding, Pulmonary hemorrhage, hematuria, hemothorax, retroperitoneal hemorrhage, and intracranial bleeding during their hospital stay was taken as the presence of bleeding complications. Patients were then followed up for 28 days post-admission via telephonic interview to look for complications such as death, readmission, thrombosis, or bleeding. If the patients were admitted to another hospital during the 28 days post-discharge, details would be collected from the discharge summary.

### Statistical analysis

The categorical variables were summarized as percentages, and continuous variables as mean (with standard deviation) or median (with interquartile range). The included patients were categorized into two groups based on whether they developed thrombosis. The two groups’ TEG parameters, clinical profiles, and outcomes were compared. The t-test was used for continuous variables, and the Chi-square test was used for categorical variables. A p-value of less than 0.05 was considered significant.

## Results

A total of 166 patients who met the inclusion and exclusion criteria between June 2021 and May 2022 were enrolled in the study. Of these, 70% were male. The median age of the patients was 61.8 years (± 15.5). The median Charlson Comorbidity score was 4 (IQR 2-6.25). The patients presented to the emergency department a median of 3 days (IQR 2-5.25) after the onset of symptoms. The mean NIAID score at admission was 4.3 (± 0.7) among the study population. One-fifth of the patients (20%) were in shock and required vasopressors upon admission, as seen in Table 1. Based on the WHO severity score, 46% of patients had critical COVID.

A total of 13 patients had regional wall motion abnormalities on the day of admission. Eleven patients had right atrium and right ventricular dilatation, and two patients had DVT on the day of admission. PT and APTT were deranged in 5 patients. Platelet count was < 1,00,000 in 36 patients. Among 166 patients, 33% had elevated D-dimer levels at admission.

**Table 1:** Demographic and clinical details at the time of admission of patients enrolled

Variables	Values [ %]
Mean Age	61.8+/-15.5
Gender	Male 117 (70%)
	Female 49 (30%)
Median onset of symptoms	3 Days (IQR 2-5.25)
**Comorbidities**	
HTN	102 (60%)
DM	101 (60%)
CKD	35 (21%)
IHD	34 (21%)
Heart failure	29 (17%)
CVA	24 (14%)
COPD	19(11%)
Asthma	8(4%)
Septic shock	35 (20%)
**WHO category at admission**	
Critical	78 (46%)
Severe	37 (22%)
Moderate	33 (19%)
Mild	18 (10%)
**NIAD score at Admission**	
3	1 (1%)
4	130 (78%)
5	19 (11%)
6	13 (7%)
7	3 (1%)
**Prior Antiplatelets usage**	
No antiplatelets	127 (76%)
Single antiplatelets	28 (16%)
Dual antiplatelets	11 (2%)
Prior Anticoagulant usage	4 (2%)

Abbreviations: HTN: Hypertension; DM: Diabetes Mellitus; CKD: Chronic Kidney Disease; IHD: Ischemic Heart Disease; CVA: Cerebrovascular Accident; COPD: Chronic Obstructive Pulmonary Disease; WHO: World Health Organization; NIAID: National Institute of Allergy and Infectious Diseases; IQR: Interquartile Range

**Fig. 1:**
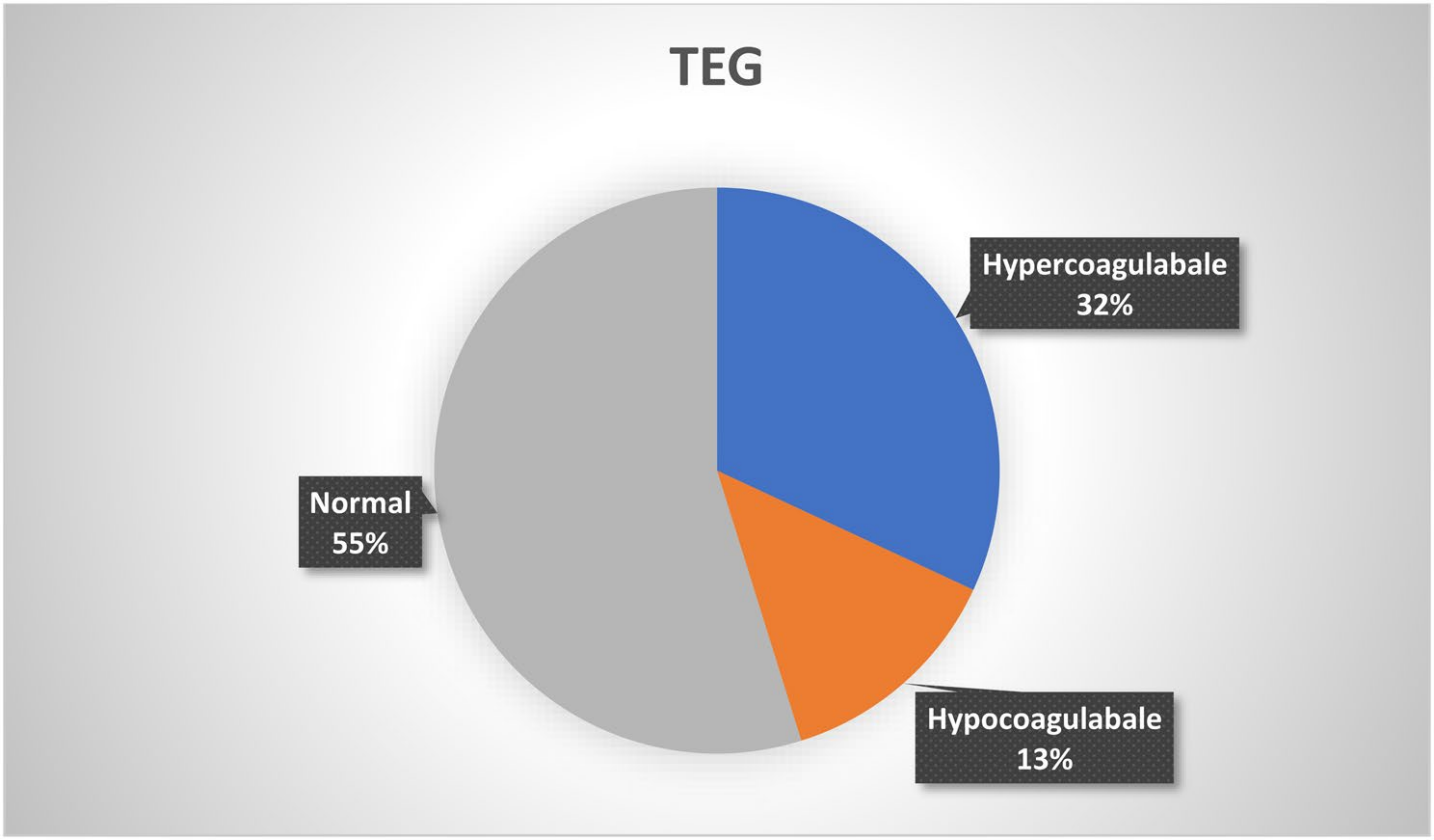
Distribution of TEG among the study population

Among 166 patients, TEG was abnormal in 75 patients (45%). Hypercoagulability was seen in 1/3rd (32%) of patients, and the TEG was hypo-coagulable in 22 patients as described in Fig. 1. In 16 patients, TEG showed fibrinolysis, among which 11 patients showed secondary fibrinolysis, and 5 showed primary fibrinolysis. Out of 15 cases showing thrombotic complications, TEG was hypercoagulable in 40% of the patients, and secondary fibrinolysis was seen in 4 patients. Anticoagulation was started for 126 out of 166 patients. Low molecular weight heparin was initiated for 76 patients, and the remaining were started on heparin. Antiplatelet drugs were started in all the patients with thrombosis and 78% without thrombosis. Among patients with previous anticoagulation and antiplatelet usage, TEG was hypercoagulable in 1 patient (1 out of 4) and 14 patients (14 out of 39), respectively. Only 4 patients with previous antiplatelet usage and 1 with previous anticoagulation usage have hypocoagulable TEG.As noted in Table 2, the TEG parameters of the COVID-19 patients were compared with the institutional reference range.

**Table 2:** TEG parameter of COVID-19 study patients with institutional reference range

	Institutional Reference value		COVID-19 study population	
Parameter	Mean [SD]	Range	Mean [SD]	Range
R time	6.22(1.77)	3.3–10.6	4.3 [1.9]	1.3–14
K Time	2.00(0.44)	1.2–3.2	1.85[1.36]	0.8–10.2
Alpha angle	61.26(5.9)	54.9–72	65.9[12.3]	4.6–85
MA	55.45(5.93)	51.2–62.6	61.8[10.4]	8.8–79.5
LY30	3.14(3.17)	0–10	4.67[10.7]	-0.2-59.8
CI	− 0.17(1.47)	-2.1-3.2	1.8[3.23]	0-3.2

Patient samples were compared with the institutional reference range

Abbreviations: R time -reaction time, MA: maximum amplitude, LY30: lysis at 30 min, CI -clot index.SD- standard deviation

As noted in Table 3, Among 166 patients, 60% were discharged from the hospital, 27% died, and 13% went against medical advice from the hospital. A telephonic follow-up was done for the patients to assess the 28-day mortality. Twenty-eight-day mortality in our study population was 32.3% (53/164, 2 were lost to follow-up). Among 166 patients, thrombotic complications were seen in 15 patients. The most common thrombotic complications were stroke (7, 4.2%), followed by myocardial infarction (6, 3.6%), pulmonary embolism (2, 1.2%), and DVT (1, 0.6%). In the study population, 19 patients had bleeding manifestations, of which 13 patients developed bleeding following initiation of anticoagulation.

**Table 3:** Outcome of study population

In-Hospital Outcomes	*n* (%)
Death	46 (27%)
Discharge	99 (60%)
Discharge against medical advice	11 (13%)
**28-day mortality**	53/164 (32.3%)
**THROMBOSIS**	
Stroke	7 (4.2%)
MI	6 (3.6%)
PE	2 (1.2%)
DVT	1 (0.6%)
**BLEEDING**	
Hematuria	6 (3.6%)
Upper GI bleed	4 (2.4%)
Pulmonary hemorrhage	3 (1.8%)
Soft tissue hematomas	2 (1.2%)
Intracranial bleed	1 (0.6%)

MI: Myocardial infarction, PE: pulmonary Embolism, DVT: Deep Vein Thrombosis, GI Bleed: Gastrointestinal Bleed

As noted in Table 4, we found that a higher total count and platelet count were associated with a higher incidence of thrombosis among the laboratory parameters. Among the patients with thrombosis, TEG was normal in 9 patients and hypercoagulable in the remaining patients. In our study, the TEG parameters did not significantly predict thrombotic complications.

**Table 4:** Factors associated with thrombotic events in COVID-19 patients

	Thrombosis (*N* = 15)	No thrombosis (*N* = 151)	*p*-value
Age	68.1 ± 11	61.1 ± 15.7	0.096
Male	12(80%)	105(69.5%)	0.397
Septic shock at admission	6(40%)	29(19%)	0.06
Comorbidities			
Diabetes	10(66.7%)	91(60%)	0.628
Hypertension	12(80%)	90(59.6%)	0.122
Chronic kidney disease	4(27.7%)	31(20.5%)	0.578
Prior stroke	2(13.4%)	22(14.5%)	0.897
Heart failure	5(33.4%)	24(15.8%)	0.09
Ischemic heart disease	5(33.4%)	29(19.2%)	0.196
Chronic obstructive pulmonary disease	2(13.4%)	17(11.2%)	0.81
Asthma	0	8(5%)	0.361
Previous COVID	0	1(0.6%)	0.752
Received prior COVID vaccination	9(60%)	78(51.6%)	0.554
**TEG at admission**			
Hypercoagulable	6(40%)	47(31.1%)	0.482
Hypocoagulable	0	22(14.5%)	0.112
Primary Fibrinolysis	1(6%)	4(2%)	0.385
Secondary Fibrinolysis	1(6%)	10(6%)	0.995
R	3.9 ± 1.2	4.4 ± 2	0.356
K	1.5 ± 0.7	1.9 *+* 1.4	0.339
Alpha	66.9 ± 7.5	65.8 ± 12.7	0.730
MA	64.6 ± 8.6	61.6 *±* 10.6	0.284
G	9.9 ± 3.8	8.9 ± 3.2	0.220
Other laboratory parameters			
TC	14,500 + 9400	10,200 ± 5800	0.011
Platelets	2.66 ± 1.1	2.1 ± 1	0.046
INR	1.15 ± 0.35	1.14 ± 0.34	0.840
aPTT	29.6 ± 5.1	32.9 ± 7.5	0.091
D-dimer	3.4 ± 3.3	2.9 ± 3.1	0.59
Outcomes			
28-day mortality	3(20%)	50(33.1%)	0.345

Abbreviations: R time -reaction time, MA: maximum amplitude, TC: Total white blood cell count, international normalized ratio, aPTT- activated partial thromboplastin time

Factors associated with 28-day mortality are analyzed and represented in Tables 5 and 6.

**Table 5:** Factors associated with death and survival on 28-day follow-up [ *n* = 164]

	Death	Survivors	*p*-value
Overall	53(35%)	111(65%)	
Age	60.83 ± 16.8	62 ± 15	0.096
Female	9(16.9%)	38(34.2%)	0.3
Septic shock at admission	19(35.8%)	16(14.4%)	0.003
**TEG at admission**			
Hypercoagulable	15(28.3%)	37(33.4%)	0.640
Hypocoagulable	12(22.6%)	9(8%)	0.019
Primary Fibrinolysis	4(7%)	1(0.9%)	0.067
Secondary Fibrinolysis	5(9%)	6(5%)	0.528
R	4.68 ± 2.3	4.19 ± 1.6	0.182
K	2.22 ± 1.7	1.67 ± 1.1	0.039
Alpha	63.3 ± 13.6	67.2 ± 11.5	0.085
MA	60.4 ± 11	62.6 ± 9.8	0.224
G	8.63 ± 3.8	9.13 ± 2.8	0.159
Other laboratory parameters			
TC	10,877	10,524	0.762
PLt	1.98 ± 1	2.22 ± 0.9	0.161
INR	1.28 + 0.49	1.08 + 0.2	0.007
aPTT	34.21 ± 7.7	31.7 ± 7.1	0.081
D-dimer	2.37 ± 2.3	1.97 ± 2	0.36

**Table 6:** Factors associated with 28-day mortality using logistic regression analysis

Parameters	Odds ratios [CI]	*P* value
TEG [Hypercoagulable]	1.33[0.51–3.45]	0.556
TEG [Hypocoagulable]	7.49[1.06–54.5]	0.042
Septic shock	2.18[0.88–5.44]	0.091
WHO category [critical]	31.85[4.59–695]	0.004
WHO Category [severe]	8.30[1.05–187]	0.083

As seen in Table 6, logistic regression analysis for the 28-day mortality shows that patients who had hypocoagulable TEG had a higher 28-day mortality with septic shock and WHO Category were taken as confounders.

## Discussion

In our observational study assessing the clinical utility of TEG in patients with COVID-19 presenting to the emergency department, we found that TEG at admission was not predictive of thrombotic or bleeding complications. The TEG profile of COVID-19 patients suggests that 32% were hypercoagulable, and 13% were hypocoagulable. The hypercoagulable picture was defined as CI >3 with either of the following: normal or shortened R and K time, increased alpha angle, and MA, and a hypo coagulable state was defined as CI < − 3 with either one of the following: prolonged R and K time, decreased alpha angle and MA, as per a previous study based on a similar population [11]. A hypercoagulable state is commonly seen in patients with COVID-19. Saseedharan et al., in their study, demonstrated an incidence of 62.5% hypercoagulable TEG in patients with moderate to severe COVID-19 admitted to the ICU [13]. Similarly, Maatman et al. had a 50% incidence of hypercoagulable TEG in patients with severe COVID-19 [14]. Panigada et al. also demonstrated a hypercoagulable state in patients intubated due to COVID-19 (raised MA in 87% and low Lys-30 in 100%) [15]. The hypercoagulable state is seen more with severe COVID-19 and may persist even in the presence of anticoagulation medication. The lower number of patients in our study with altered coagulation profiles as identified with TEG could be due to the following reasons: (a) we had a mix of both moderate and severe COVID-19, (b) since only one TEG value was sent on the day of admission, (median 3 days since onset of symptoms), we may have missed any ongoing changes in TEG during the illness.

We had a higher incidence of primary and secondary fibrinolysis (3% and 6%). It was similar to the study by Pazzi et al. showed that hypo fibrinolysis rather than hypercoagulability seems to be associated with poor and adverse outcomes [16]. Incidence of thrombotic events in our study was 9%, which was similar to Lodigiani et al. i.e., 7.7% [17]. Our study showed that common thrombotic complications were stroke, followed by myocardial infarction, pulmonary embolism, and DVT, which was similar to the systematic review by Hartmann et al. 2022 [9].

We didn’t find any statistically significant correlation between the incidence of thrombosis and the presence of a hypercoagulable state on TEG. The use of TEG to predict thromboembolism or mortality is not yet fully proven. Salem et al. also found no correlation between a hypercoagulable state and the incidence of thromboembolic phenomenon [10]. Duenas et al., in their retrospective study on 134 critically ill COVID-19 patients, found no benefit of TEG-guided anticoagulation protocol in reducing death, major bleeding, or thrombosis [18]. Marvi et al. found that low Maximum Amplitude (MA) in serial TEG measurements in critically ill COVID-19 patients was associated with a higher risk of venous thromboembolism [19]. Similarly, Zhong et al., in their observational study, found an increased Maximum Amplitude to be associated with higher mortality [20]. 

There could be many reasons why we didn’t find any significant correlation between Thrombotic events and TEG parameters. Firstly, we only measured TEG at admission. Secondly, most patients were on prophylactic or therapeutic anticoagulation during their admission. Also, we included both arterial and venous thromboembolic complications as a single composite, even though they have different pathologies due to their similar clinical implications.

With regards to bleeding complications, we had 16 patients (9.6%) with various complications such as Upper GI bleed, pulmonary hemorrhage, and Intracranial bleeding. In a recent systematic review and meta-analysis of 49 studies and >18,000 patients by Jiménez et al., there was a pooled incidence of 7.8% (95% CI, 2.6–15.3) for bleeding and 3.9% (95% CI, 1.2–7.9) for significant bleeding [21]. The cause of bleeding could be primarily due to COVID-19-associated coagulopathy or due to confounding factors such as the use of anticoagulants, steroids, and prolonged ICU stay. Similarly, Parks et al., in their observational study, found 5-fold increase in the risk of bleeding secondary to anticoagulation [22].

Our study has shown that there was a correlation between the Hypocoagulable TEG [ odds ratio 7.49] with the mortality at 28-day mortality, which was similar to other studies such as Mohan et al. [12] and Blasi et al. [23]. Multivariable Logistic regression analysis about the factors affecting mortality showed that hypo-coagulability was 7.49 times affect mortality, which was similar to Al Sankari et al. [24]. Kong et al. [26] and Adamzink et al. [25] showed that TEG showing hypo-coagulable and septic shock showed increased mortality. As the disease progressed, TEG profiles of the COVID-19 patients shifted towards hypo-coagulability, which was associated with poor prognosis and increased mortality. The proposed hypothesis for the hypocoagulable TEG in some cases of Severe COVID-19 is due to reduced thrombin generation, which results in COVID-associated coagulopathy [CAC]. It is considered similar to the development of coagulopathy in other consumptive coagulopathies like sepsis-induced coagulopathy [5, 8, 15]. Patients with severe metabolic acidosis and septic shock also have reduced thrombin generation. As the disease severity of COVID-19 progresses, it will lead to a stage of complete cellular shutdown [27]. Further studies are required to confirm this proposed hypothesis.

The implications of sending TEG early helps in deciding the requirement of anticoagulation dose starting based on case to case basis. Findings from a randomised control trial on the effect of anticoagulation dose in patients with severe or critically ill patients showed that there were increased bleeding manifestations in critically ill patients and the enrolment of the critically ill patients were suspended [28]. Hypocoagulable TEG and anticoagulation dose were to be decided on the risk vs. benefit ratio and further studies were required.

This study was one of the few prospective studies looking for venous and arterial thrombotic and bleeding complications in COVID-19 patients. We also tried to assess the benefits of sending TEG early, directly from the emergency department as a point-of-care test, which was not done previously, as the remaining studies were done in the ICU. There are a few limitations to our study. Ours was a single-center observational study, limiting its generalizability to larger populations. The COVID variant was not identified in our patients. Different variants of the COVID-19 virus could have other types of effects on the coagulation and might have confounded our findings. Patients on antiplatelet or anticoagulant therapy were not excluded from the study.

## Conclusion

COVID-associated coagulopathy can be associated with both thrombotic (venous and arterial) and bleeding complications. Hypocoagulability on initial TEG has a 7.49 times increased risk of mortality. Hypocoagulable TEG indicates an early predictor for mortality. Early TEG can be an essential tool in managing COVID-associated coagulopathy.

## Supplementary Information

Below is the link to the electronic supplementary material.

Supplementary Material 1

## Data Availability

Data is provided in the Manuscript as supplementary material.

## Abbreviations

COVID: Coronavirus disease
TEG: Thromboelastography
SARS-CoV-2: Severe Acute Respiratory Syndrome Coronavirus 2
CAC: COVID-19-associated coagulopathy
HTN: Hypertension
DM: Diabetes Mellitus
CKD: Chronic Kidney Disease
IHD: Ischemic Heart Disease
CVA: Cerebrovascular Accident
COPD: Chronic Obstructive Pulmonary Disease
WHO: World Health Organization
NIAID: National Institute of Allergy and Infectious Diseases
R time: Reaction time
MA: Maximum amplitude
LY30: Lysis at 30 min
CI: Clot index
MI: Myocardial infarction
PE: Pulmonary Embolism
DVT: Deep Vein Thrombosis
GI Bleed: Gastrointestinal Bleed
INR: International normalized ratio
aPTT: Activated partial thromboplastin time
